# *Afzelia corallina* (Fabaceae), a new micro-endemic tree from Pemba Island, Zanzibar, Tanzania

**DOI:** 10.3897/phytokeys.273.186903

**Published:** 2026-04-09

**Authors:** Andrea Bianchi, Giacomo Baldesi, Daniela Calzoni, Massimo Delledonne, Riccardo Focaia, Quentin Luke, Khamis A. Khamis, Laura Tomasi, Simone Orsenigo

**Affiliations:** 1 Istituto Oikos, Via Crescenzago 1, 20134, Milano, Italy MUSE – Museo delle Scienze Trento Italy; 2 MUSE – Museo delle Scienze, Corso del lavoro e della scienza 3, 38122, Trento, Italy University of Pavia Pavia Italy; 3 Department of Earth and Environmental Sciences, University of Pavia, Via Sant’Epifanio 14, 27100, Pavia, Italy University of Verona Verona Italy; 4 Department of Biology, Centro Studi Erbario Tropicale (Herbarium FT), University of Florence, Via La Pira 4, 50121, Firenze, Italy University of Florence Firenze Italy; 5 Functional Genomics Lab, Department of Biotechnology, University of Verona, Via Albere 17, 37138, Verona, Italy National Museums of Kenya Nairobi Kenya; 6 East African Herbarium, National Museums of Kenya, P.O. Box 40658, Nairobi, Kenya Istituto Oikos Milano Italy; 7 Department of Forestry, P.O. Box 283 Wete, Pemba Island, Zanzibar, Tanzania Department of Forestry Wete Tanzania

**Keywords:** Afzelieae, Detarioideae, new species, Ngezi, taxonomy, Vumawimbi

## Abstract

*Afzelia corallina* A.Bianchi, S.Orsenigo & Baldesi (Fabaceae: Detarioideae) is described from the coral rag forests of the Tondooni Peninsula in the Ngezi–Vumawimbi Forest Reserve on Pemba Island, Zanzibar, Tanzania. Morphologically distinct from all known members of the genus, this large, emergent tree shows the closest affinities to *Afzelia quanzensis* but differs in several vegetative and floral characters, including its falcate leaflets, highly branched terminal inflorescences, petal colour, the colour of stamens, and the number of extra staminodes. The species is currently known only from a restricted coastal limestone habitat, indicating a micro-endemic distribution. An updated diagnostic key to East African *Afzelia* species is provided. This discovery highlights the botanical uniqueness and conservation importance of Pemba’s remaining forest ecosystems.

## Introduction

### The genus *Afzelia*

The genus *Afzelia* Sm. (Fabaceae, subfamily Detarioideae) comprises 12 species (POWO 2026) of medium to large trees distributed chiefly across tropical Africa and, to a lesser extent, Southeast Asia, inhabiting dry forests, woodland ecosystems, lowland rainforests, and thickets (Brummitt et al. 2007; Estrella et al. 2018; Gilbert and Boutique 1952). In Africa, species such as *Afzelia africana* Sm. ex Pers. and *Afzelia quanzensis* Welw. are ecologically and economically significant, valued for their durable hardwood and ecological role in woodland canopies (Donkpegan et al. 2014). At least five species are considered of commercial importance (*A. africana*, *A. bella* Harms, *A. bipindensis* Harms, *A. pachyloba* Harms, and *A. quanzensis*) and are regarded as being in high demand for international trade in their timber, raising concern about unsustainable harvesting (Cunningham 2016), hence the recent inclusion of all African *Afzelia* spp. in CITES appendix II (CITES 2022, 2025).

East Africa is home to only three *Afzelia* species (i.e. *A. africana*, *A. bipindensis*, *A. quanzensis*), of which *A. quanzensis* is the most widespread, occurring from coastal Somalia, Kenya, and Tanzania south to northern South Africa, including inland regions from Mozambique to Angola (Brenan 1967; Brummitt et al. 2007).

The genus *Afzelia* belongs to the tribe Detarieae*s.l*. within the subfamily Detarioideae, an early-diverging lineage of tropical trees characterised by complex floral morphology and strong biogeographic structuring (Estrella et al. 2018). The *Afzelia* clade, as circumscribed by Bruneau et al. (2008) and now recognised as the tribe Afzelieae, shows a striking biogeographical pattern, comprising three genera with disjunct distributions. *Brodriguesia* R.S.Cowan is monotypic and restricted to the Atlantic Forests of Brazil; *Afzelia* is predominantly African and is hypothesised to have originated in savanna environments, although it also includes polyploid species occurring in forest habitats (Donkpegan et al. 2020). *Intsia* Thouars exhibits a wide distribution spanning both sides of the Indian Ocean and the West Pacific and is thought to have achieved this range through oceanic dispersal (Estrella et al. 2018).

### Pemba Island

Pemba is an oceanic island that lies approximately 50 km off the northern coast of Tanzania and, together with Unguja, forms the Zanzibar Archipelago. The principal forest on the island is the Ngezi–Vumawimbi Nature Forest Reserve. It is situated in the northern part of the island; it covers 2031 ha (Silvia Ceppi, Oikos, pers. comm.) and constitutes the largest extant tract of old-growth coastal and moist forest within the archipelago. This forest reserve comprises a diverse assemblage of natural habitats, including lowland moist forest, riverine and coastal forest, *Erica mafiensis* (Engl.) Dorr heathland, mangrove stands, and coral rag vegetation (Beentje 1990; Nahonyo et al. 2005; Orsenigo et al. 2026b).

Pemba Island is known for its high level of endemism and floristic distinctiveness compared to the mainland and even neighbouring Unguja Island, a pattern attributed to its unique geological history and long-term isolation (Beentje 1990; Orsenigo et al. 2026b). The Ngezi–Vumawimbi Forest Reserve represents one of the last remaining fragments of Pemba’s original forest cover and has recently yielded several new plant species discoveries for the island (Orsenigo et al. 2026a) and for science (Orsenigo et al. 2026b), highlighting its significance as an area of high biodiversity concentration.

During recent botanical surveys of the reserve, a distinctive population of *Afzelia* was discovered in the coral rag forests of the Tondooni Peninsula. This taxon shows clear morphological affinities with *A. quanzensis* but differs markedly in several vegetative and floral characters, including leaf arrangement, inflorescence architecture, petal colouration, and flower morphology. Despite extensive comparisons with regional floras (Léonard 1952; Brenan 1967; Hutchinson and Dalziel 1972), herbarium material, and the two published checklists of Ngezi Forest Reserve (Beentje 1990; Nahonyo 2005), the taxon could not be matched to any known species.

*A. quanzensis* was the only species previously reported from Pemba (Brenan 1967; Beentje 1990), together with the related *Intsia bijuga* (Colebr.) Kuntze, which only in recent surveys has been confirmed for the Ngezi–Vumawimbi Nature Forest Reserve (Orsenigo et al. 2026b), forming dense stands in the NW part of the reserve and small patches in the south.

Here, we describe this new *Afzelia* taxon as a distinct species and provide detailed morphological and genetic comparisons with its closest related species, *A. quanzensis*. We also present an updated key to the East African species of *Afzelia* and discuss the new species’ ecology, conservation status, and biogeographic implications.

## Methods

### Morphological analysis

Field surveys were carried out in the Ngezi–Vumawimbi Nature Forest Reserve across various field visits between December 2024, when this new taxon was first observed, and January 2026 (by A. Bianchi, S. Orsenigo, G. Baldesi, K. Khamis and Q. Luke). The description of this new species of *Afzelia* is based on direct measurements in the field as well as on herbarium specimens (collected with permission granted by the Department of Forestry of Zanzibar) preserved at NHT, EA, FT, PAV and TR. Abbreviations follow Thiers (2026).

Photographs were taken using a mirrorless camera (Canon R50) equipped with a macro lens (Canon EF 100 mm f/2.8L Macro IS USM). Measurements of leaves, twigs, seeds, pods and flowers were taken using a digital calliper [RS PRO, Digital Calliper 6” (150 mm) ± 0.001” (0.03 mm) accuracy LCD stainless steel RoHS]. Measurements of tree heights were made using a rangefinder (Nikon Forestry III).

Morphological comparisons were made with the closest related species, *A. quanzensis*, based on herbarium specimens housed at NHT, EA, FT, MO and K. Plant terminology follows Beentje (2010), and nomenclature follows the “International Code of Nomenclature for algae, fungi and plants” (Turland et al. 2025).

### DNA extraction and WGS sequencing

Genomic DNA (gDNA) was extracted from 1 g of silica gel–dried leaf tissue using the cetyltrimethylammonium bromide (CTAB) protocol (Carlson et al. 1991). The integrity of the extracted gDNA was assessed using a TapeStation 4150 system (Agilent Technologies, Inc., Santa Clara, CA, USA), while DNA concentration was quantified with a Qubit 4 Fluorometer (Thermo Fisher Scientific, Waltham, MA, USA). To remove short DNA fragments, the gDNA sample was purified using 0.4× AMPure XP beads (Beckman Coulter Inc., Brea, CA, USA). A total of 260 ng of purified gDNA was mechanically fragmented using a Covaris S220 system under the following conditions: 55 s treatment time, 6 °C, peak incident power of 75 W, duty factor of 10%, and 1000 cycles per burst. The fragmented gDNA was subsequently processed using the KAPA HyperPrep PCR-free library preparation kit (Roche, Basel, Switzerland) according to the manufacturer’s instructions to generate the final sequencing library. The library was sequenced on an Illumina NovaSeq X platform (Illumina, Inc., San Diego, CA, USA) using paired-end 150 bp reads, generating approximately 70 million fragments.

### Plastome assembly and phylogenomic analysis

The chloroplast genome was assembled using GetOrganelle v1.7.7.1 (Jin et al. 2020), and its structure was visually inspected with Bandage (Wick et al. 2015). To assess its phylogenetic relationships with related species, we selected 28 species within the family Detarioideae. This included specimens from the tribes Barnebydendreae, Detarieae, Saraceae, Afzelieae, and Amherstieae. Whole chloroplast sequences were aligned using MAFFT (Katoh et al. 2002) with default parameters. We constructed a maximum likelihood phylogenetic tree using IQ-TREE2 with 1,000 bootstrap replicates and an automatic model of substitution (Minh et al. 2020). *Quillaja saponaria* Molina (MN709839.1) was used as an outgroup following the approach described in U-thoomporn et al. (2022).

## Results

### Morphological comparison

Morphological differences between *Afzelia quanzensis* and *Afzelia corallina* sp. nov. are summarised in Table 1.

**Table 1.** Morphological differences between *Afzelia quanzensis* and *Afzelia corallina* sp. nov.

	*Afzelia quanzensis*	*Afzelia corallina* sp. nov.
**Twigs**	sometimes moderately pubescent, glabrescent	glabrous
**Leaflets size**	2.3–12 × 1.5–7.2 cm	(5–) 9–12 (–16) × (4.5–) 6.5–7.5 cm
**Leaflets shape**	broadly ovate to almost elliptic	ovate to oblong, tending to falcate
**Base**	cuneate to rounded	rounded
**Inflorescence**	simple or one forked	panicle
**Inflorescence**	4–12 flowered	up to 150–flowered
**Hypanthium**	11–25 mm long	15–30 mm long
**Pedicel**	ca. 5 mm	7–8 mm
**Sepals indumentum**	puberulous outside, glabrous inside	puberulous outside and puberulous to sparsely pubescent inside
**Outer sepals**	elliptic, 9–17 × 7–13 mm	elliptic to ovate, 12–15 × 6–7 mm
**Inner sepals**	obovate-spathulate, 17–25 × 9–18 mm	oblong, but slightly wider distally 17–18 × 7–8 mm
**Large petal**	green outside, red inside and occasionally mottled with white or greenish-white	light-pink outside, white and red inside, with a white median stripe
**Claw size**	up to 2 cm long	0.8–1 cm long
**Large petal indumentum**	pubescent	pubescent adaxially from base to widening of the lamina or shortly after, puberulous abaxially for ⅔ of petal length
**Small petals**	4, clavate	4, greenish-white, shortly pubescent, subulate, 6–9 × 0.5 mm
**Fertile stamens**	(5–) 7 (–9,) green and often red at the base, pilose near the base or glabrous, 35 mm long	7, crimson red, pilose proximally to sparsely pilose and eventually glabrous in last ¼, 30–40 mm long,
**Staminodes**	usually 2, green	usually 2, crimson red
**Extra staminodes**	no	sometimes two, minute, green
**Ovary**	ca. 3.5 × 1.5 mm, pilose particularly on the margins	ca. 7 × 2.2–2.7 mm, glabrous to shortly pubescent
**Style**	25 mm	40–50 mm
**Pod shape**	straight, 7–23 × 4.5–8.3 cm	straight, 7–13 × 4.5–6 cm
	5–13-seeded. Woody, each valve up to 2 cm thick.	(1–) 2–3 (-4) seeded.
		thinly woody, each valve up to 5 mm thick.
**Seeds**	ellipsoid, 20–34 × 9–17 mm	flattened dorsiventrally, 25–32 × 12–14 mm, 6 mm thick
**Aril**	reddish orange, cup shaped, 8–13 mm long, covering 1/4 to 1/3 of the seed-	yellowish orange, cup shaped, thin, 4–5 mm long, covering less than 1/5 of the seed.

### Phylogenomic analysis

The assembled plastid genome resulted in the typical circular DNA structure of 159,388 bp, with a large single-copy region (LSC) and a smaller one (SSC) with 88,526 and 19,824 bp, respectively, separated by an inverted repeat region (IR) of 25,729 bp.

**Figure 1.**
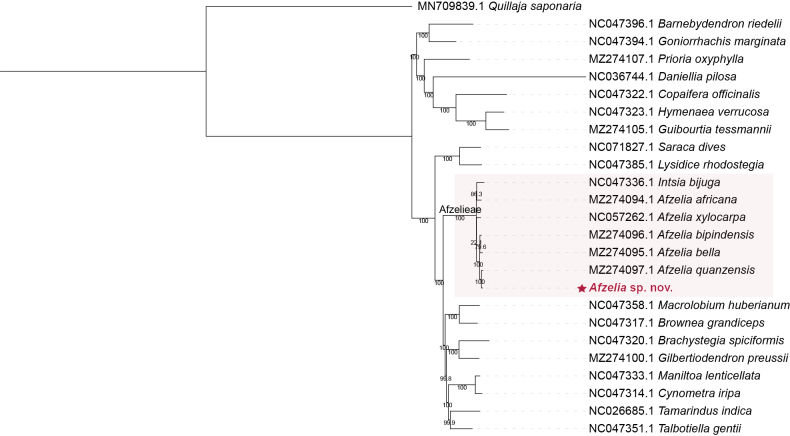
Phylogenetic tree of plastomes of Detarioideae, constructed using maximum likelihood with IQ-TREE2 with 1,000 bootstrap replicates and an automatic model of substitution. *Quillaja saponaria* is used as an outgroup.

The ML phylogenetic tree is consistent with recent studies on related taxa within Detarioideae (Donkpegan et al. 2017; Estrella et al. 2020). It confirms the monophyly of the tribe Afzelieae, which includes the genera *Intsia* and *Afzelia*. *Afzelia corallina* sp. nov. is recovered as closely related to the genus *Afzelia*, with 100% bootstrap support (Fig. 1).

### Taxonomic treatment

#### Afzelia corallina

Taxon classificationPlantaeFabalesFabaceae

A.Bianchi, S.Orsenigo & Baldesi sp. nov.

97E13C52-554C-5B0C-B795-DBFD87D160D4

urn:lsid:ipni.org:names:77374530-1

Figs 2, 3, 4, 5

##### Type.

Tanzania • Zanzibar, Pemba Island, Micheweni Dist., Ngezi-Vumawimbi Nature Forest Reserve, coral rag forest in Tondooni Peninsula; -4.94073, 39.67400; 10 December 2024; *Bianchi, Orsenigo & Baldesi 476* (holotype, NHT!; Isotypes, EA!, FT!, PAV!, TR!)

**Figure 2.**
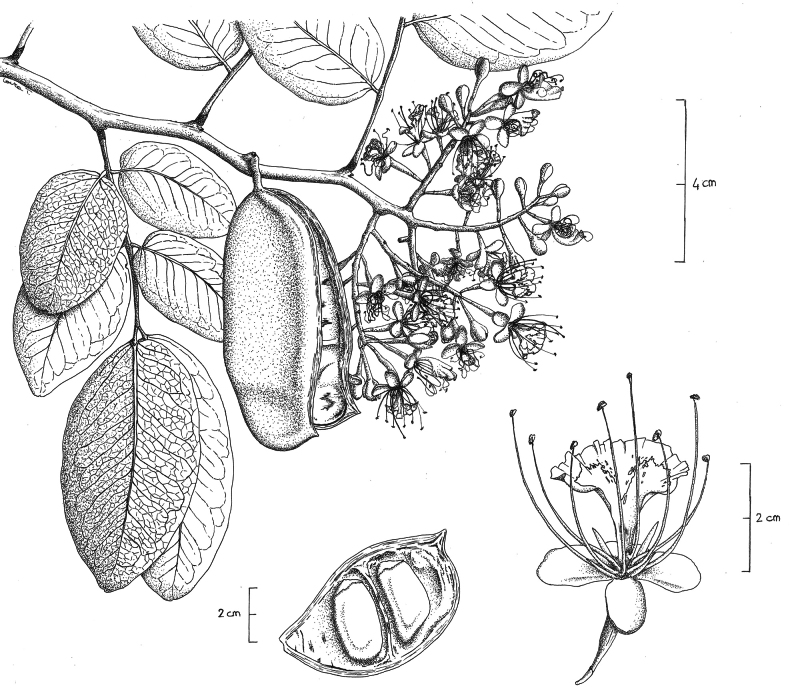
Illustration of *Afzelia corallina*. **a**. Flowering branch, showing leaves, ripe pod with seeds dispersed and inflorescence; **b**. Flower details; **c**. Pod valve showing arillate seeds. Drawn by: Laura Tomasi.

##### Diagnosis.

This species is similar to *Afzelia quanzensis* but can be distinguished by paniculate inflorescence (vs. racemose or one-forked inflorescence), number of flowers per inflorescence (up to 150 vs. 4–12), colour of the large petal (white and red, with a white median stripe vs. entirely green outside and red inside, occasionally mottled with white or greenish-white), the shape of small petals (subulate vs. clavate), the colour of stamen and staminodes that are crimson red in *A. corallina* and green, often with a red base, in *A. quanzensis*. Moreover, *A. corallina* shows a bigger style (40–50 mm vs 25 mm) and ovary (7 × 2.2–2.7 vs. 3.5 × 1.5 mm) compared to *A. quanzensis*. Finally, pods are thinly woody and 1–4 seeded (vs. thickly woody and 5–13 seeded in *A. quanzensis*), and seeds have much smaller aril (4–5 vs. 8–13 mm long) (Table 1).

**Figure 3.**
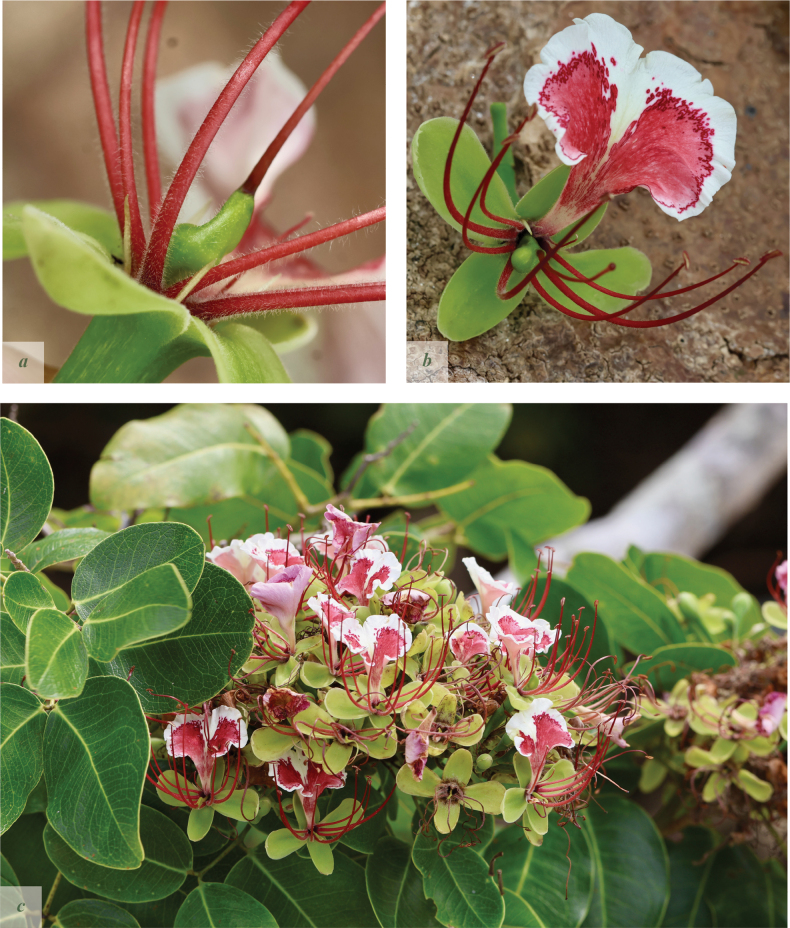
*Afzelia corallina*. **a**. Flower close-up showing two rudimentary subulate petals; **b**. Single flower; **c**. Inflorescence. Pictures by: Andrea Bianchi.

##### Description.

Large tree up to 20 (–25) metres tall, with spreading crown, emergent above surrounding vegetation; bole straight, cylindrical, unbranched for the first (2–) 8–10 metres, up to 1.1 m in diameter, not conspicuously buttressed; wood heavy, durable, dark brown to reddish, with yellowish pores; bark light brown with white, lichen-like patches, covering 10–30% of bark surface on older wood and up to 80% on smaller branches; bark scaling off in patches in the lower part of the bole, otherwise smooth; twigs glabrous, with longitudinal striations and numerous lenticels, ca. 1 × 1 mm. Stipules not seen; leaves dark green with yellowish venation, glabrous in all parts, paripinnate; leaflets (2–) 3–4 pairs, opposite to sub-opposite; leaf petiole 2–3 cm long, rachis 8–10 cm long, petiolules 3.5–5.5 mm long, twisted, with one side of the leaf base slightly decurrent down the petiolule for up to 2.5–3 mm; leaflets (5–) 9–12 (–16) cm long, (4.5–) 6.5–7.5 cm wide, ovate to oblong, tending to (slightly) falcate in larger leaflets, especially in distal pair; leaflets base rounded and asymmetrical at base, leaflet tip rounded to acute distally with emarginate apex; venation pinnate, with secondary veins clearly visible and raised in both adaxial and abaxial leaf surfaces, brochidodromous, tertiary venation reticulated. Inflorescence terminal, a corymbose panicle branched up to 7 times or more, up to 150-flowered; inflorescence rachis and primary branches puberulous; pedicel 7–8 mm long, bracteoles not seen. Flowers (very) sweetly scented; hypanthium 1.5–2.5(–3) cm long, puberulous; sepals light green to yellowish in older flowers, puberulous on outside and puberulous to sparsely pubescent inside, the outer two elliptic to ovate, 12–15 × 6–7 mm, the inner 2 oblong but slightly wider distally, 17–18 × 7–8 mm wide; large petal turned upwards, suffused with light-pink abaxially (paler at anthesis and darkening as the flower ages) and bicoloured adaxially, pure white with a conspicuous crimson red bilobed marking in the centre speckled with darker spots at its edges, (24–) 28–33 mm long, with an 8–10 mm long claw widening into a narrowly bilobed lamina 20–25 mm wide; large petal pubescent adaxially from base to widening of the lamina or shortly after, puberulous abaxially for ⅔ of petal length; small petals 4, greenish-white, shortly pubescent, subulate, 6–9 × ca. 0.5 mm; fertile stamens 7, crimson red, pilose proximally to sparsely pilose and eventually glabrous in last ¼, 3–4 cm long, 0.5–1 mm in diameter, at the base; anthers dorsifixed, red with two longitudinal openings revealing yellow pollen, 2.5–3.5 × 0.7–0.8 mm; staminodes 2 at base of large petal, crimson red, pubescent, 13–15 mm long, at the base of large petal, some flowers with 2 extra staminodes, minute, green, puberulous, up to 5 mm long, in between the longer staminodes; ovary green, glabrous to shortly pubescent, 7 × 2.2–2.7 mm long, style glabrous, crimson red, 4–5 cm long. Stigma capitate, ca. 1 mm in diameter. Pods woody, brown outside and tan inside, 7–13 × 4.5–6 cm, beaked distally, (1–)2–3(–4) seeded; valve ca. 3 mm thick, increasing to 5–6 mm near the sutures, septa not always present; seeds black, flattened dorsiventrally, 25–32 mm long × 12–14 mm wide × 6 mm thick, with a small, yellowish-orange thin aril covering the seed for 4–5 mm.

**Figure 4.**
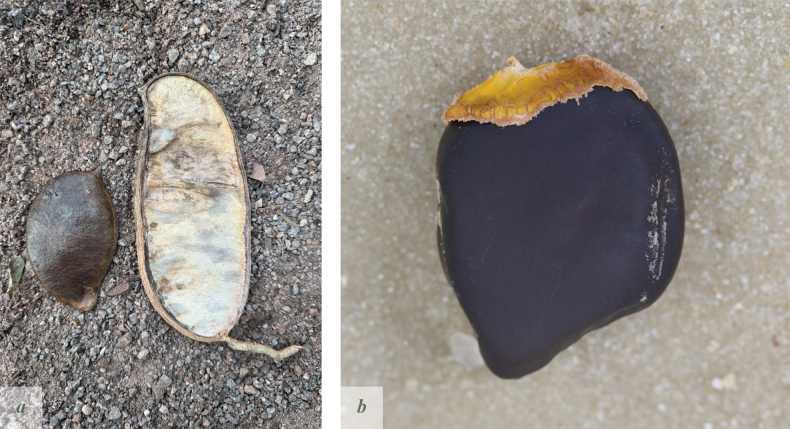
*Afzelia corallina*. **a**. Two examples of pods, 1–seeded, left, and 3–seeded, right. Note the almost complete lack of septa; **b**. Seed, showing the small, thin aril. Pictures by: Andrea Bianchi.

**Figure 5.**
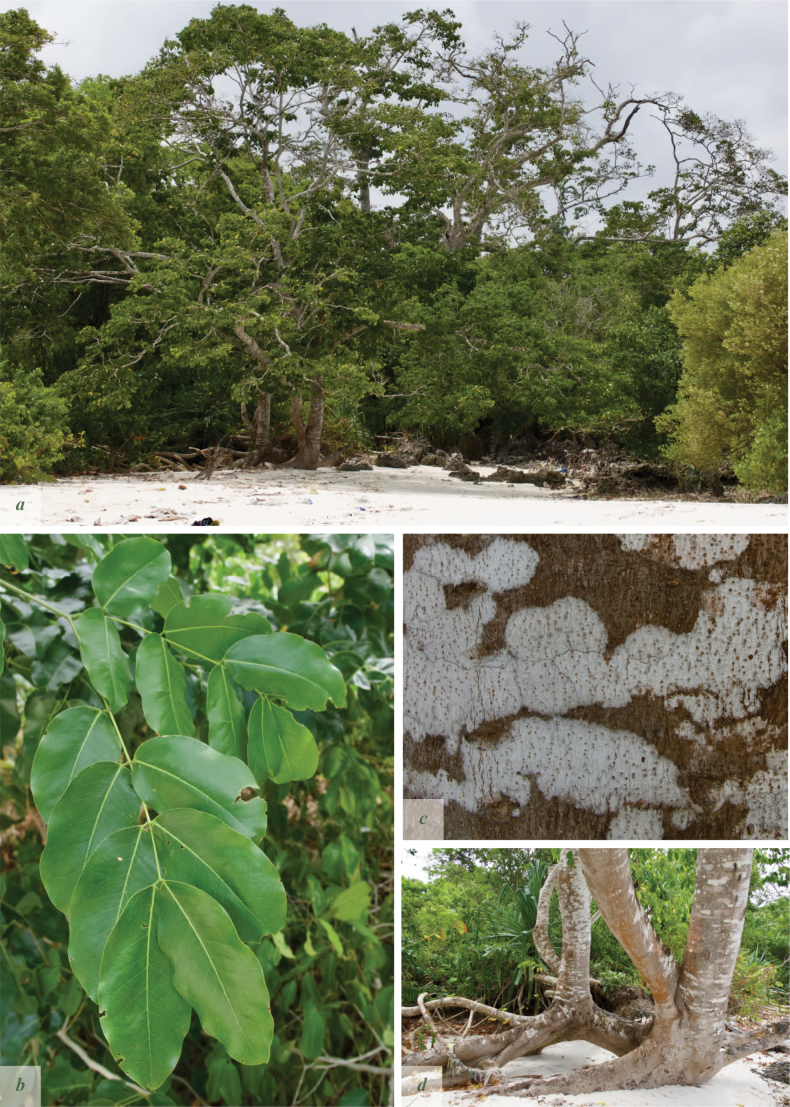
*Afzelia corallina*. **a**. Trees in habitat; **b**. Leaves; **c**. Lichen-like pattern on bark; **d**. Boles. Pictures by: Andrea Bianchi.

##### Phenology.

Found in flower in November–January, seeds dispersed in September–November.

##### Etymology.

The epithet *corallina* (from the Latin ‘*corallium*’, coral) refers to this species’ habit of growing on coral rag, a rubbly limestone composed of ancient coral reef material. Furthermore, the colourful and dense inflorescence may resemble a coral head, as does the red marking on the large petal.

##### Distribution.

Despite extensive searches in the area, *Afzelia corallina* is only known from a narrow stretch of coast in the Tondooni Peninsula in SW Ngezi-Vumawimbi Forest Reserve, Pemba Island, Tanzania.

##### Ecology.

This species is strictly associated with coastal forests and apparently does not occur more than 200 metres away from shore. It is associated with the following *taxa*; trees: *Avicennia marina* (Forssk.) Vierh. (Acanthaceae), *Bourreria petiolaris* (Lam.) Thulin (Boraginaceae), *Deinbollia borbonica* Scheff. (Sapindaceae), *Euphorbia tirucalli* L. (Euphorbiaceae), *Grewia capitellata* Bojer (Malvaceae), *Haplocoelum inoploeum* Radk. (Sapindaceae), *Manilkara sulcata* (Engl.) Dubard (Sapotaceae), *Milicia excelsa* (Welw.) C.C.Berg (Moraceae), *Pandanus kirkii* Rendle (Pandanaceae), *Polysphaeria parvifolia* Hiern (Rubiaceae), *Rhizophora mucronata* Lam. (Rhizophoraceae), *Sonneratia alba* Sm. (Lythraceae), *Tamarindus indica* L. (Fabaceae), *Xylocarpus granatum* J.Koenig (Meliaceae), climbers: *Uvaria lucida* Benth. subsp. lucida (Annonaceae), *Salacia elegans* Welw. ex Oliv. (Celastraceae), *Ancylobothrys petersiana* (Klotzsch) Pierre (Apocynaceae), *Landolphia kirkii* Dyer (Apocynaceae), *Flagellaria guineensis* Schumach (Flagellariaceae), shrubs: *Acridocarpus zanzibaricus* A.Juss (Malpighiaceae). *Indigofera trita* L.f. (Fabaceae), *Cremaspora triflora* (Thonn.) K.Schum. subsp. *confluens* (K.Schum.) Verdc. (Rubiaceae), herbs: *Microsorum scolopendria* (Burm.f.) Copel. (Polypodiaceae), *Sansevieria kirkii* Baker var. *pulchra* N.E.Br (Asparagaceae) and the bulbous *Gonatopus boivinii* (Decne.) Engl. (Araceae).

##### Conservation status.

*Afzelia corallina* is currently known only from its type locality, where fewer than 30 mature individuals have been recorded. Although this locality lies within the Ngezi–Vumawimbi Forest Reserve, illegal extraction of hardwood remains a persistent threat. This species, together with *Intsia bijuga*, *Milicia excelsa*, and *Erythrophleum suaveolens* (Guill. & Perr.) Brenan, is specifically targeted by illegal loggers. A concerning number of individuals, particularly those occurring along the coast, exhibit poor crown condition and apparent dieback. Likely, this distribution is due to the extraction of most of the straight trees occurring farther inland, while the individuals growing along the coast might have been spared because of the misshapen boles. Based on our observations, the optimum habitat for this species is behind the salt spray zone. In addition, seed production appears to be extremely low, with only a few seeds produced each year. Regeneration by seed appears poor to absent, with young individuals observed resulting from suckering from stressed mature plants growing directly along the coast. Given its restricted distribution and threats due to illegal logging and touristic infrastructure development (Caro et al. 2025), *A. corallina* should be assessed as Critically Endangered (CR). It clearly meets criteria B2ab(iii, v); D (extent of occurrence <100 km^2^ and presence at a single threat-defined location, with continuing decline in the area, extent, and quality of habitat and number of mature individuals; fewer than 50 mature individuals) (IUCN 2012). Propagation and *in situ* and *circa situ* replanting efforts are underway as part of a project funded by Fondation Franklinia and implemented by Istituto Oikos. Despite the threats posed by touristic infrastructure development, carefully managed ecotourism might represent a complementary conservation opportunity: Oikos, the NGO that partly funded this research, has been promoting sustainable ecotourism in Ngezi–Vumawimbi and mapping individuals of particular interest that could be incorporated into guided visits, hopefully generating local incentives for habitat protection. Worryingly, felling remains a threat, and one individual has been found logged during the last survey, with two additional trees found felled by recent storms.

##### Notes.

**Figure 6.**
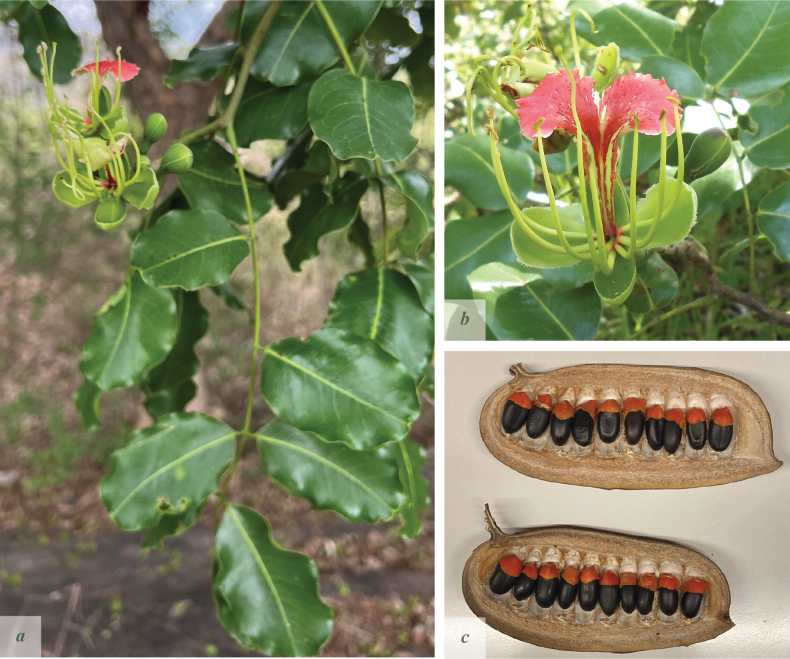
*Afzelia quanzensis*. **a**. Inflorescence and leaves; **b**. Flower; **c**. Open pods with seeds from both valves. Pictures by: Andrea Bianchi, Quentin Luke & Margherita Rinaldi, based on specimens from eastern Tanzania.

This species shows some affinities with *Intsia bijuga*, especially in the congested inflorescence, the reddish stamen, and the thinly woody pod, with few flattened seeds. It is interesting to note that across the whole range of *Afzelia quanzensis* (Fig. 6) and *Intsia bijuga*, Pemba Island is the only place where both species coexist. Further molecular analyses will help to shed light on the origin of this species.

The three Detarioideae occurring on the island are not distinguished by local people and are referred to, in Swahili, by a single name, ‘*Mbambakofi*’.

### Key to the East African species of *Afzelia*

**Table:** 

1	Aril cup-shaped; pods straight; rudimentary petals present	**2**
–	Aril markedly bilobed; pods curved-reniform; rudimentary petals absent	***A. bipindensis***
2	Hypanthium 0.3–0.6 cm long; large petal 1.1–2 cm long	***A. africana***
–	Hypanthium 1.5–3 cm long; petal 2.5–4.5 cm long	**3**
3	Inflorescence simply racemose or once-forked with 4–12 flowers; large petal green outside, red inside, only occasionally mottled with white or greenish-white; fertile stamens and staminodes green (stamens often with a red base); small petals clavate; pod thickly woody, 5–13 seeded; seeds ellipsoid with aril 8–13 mm long	***A. quanzensis***
–	Inflorescence paniculate with up to 150 flowers; large petals white and red, with a white median stripe; fertile stamens and staminodes crimson red; small petals subulate; pod thinly woody, 1–4 seeded; seeds flattened dorsiventrally with aril 4–5 mm long	***A. corallina***

## Supplementary Material

XML Treatment for Afzelia corallina

## Additional information

### Use of AI

No use of AI was reported.

### Author ORCIDs

Andrea Bianchi https://orcid.org/0000-0002-7756-2066

Giacomo Baldesi https://orcid.org/0000-0003-4441-0581

Daniela Calzoni https://orcid.org/0009-0000-8165-0491

Massimo Delledonne https://orcid.org/0000-0002-7100-4581

Riccardo Focaia https://orcid.org/0009-0001-4829-0356

Quentin Luke https://orcid.org/0000-0002-7256-031X

Khamis A. Khamis https://orcid.org/0009-0009-1148-4112

Laura Tomasi https://orcid.org/0009-0001-8568-6544

Simone Orsenigo https://orcid.org/0000-0003-0348-9115

### Data availability

All of the data that support the findings of this study are available in the main text.
